# Ultra-High-Frequency Ultrasonography of Labial Glands in Pediatric Sjögren’s Disease: A Preliminary Study

**DOI:** 10.3390/diagnostics13162695

**Published:** 2023-08-16

**Authors:** Edoardo Marrani, Giovanni Fulvio, Camilla Virgili, Rossana Izzetti, Valentina Dini, Teresa Oranges, Chiara Baldini, Gabriele Simonini

**Affiliations:** 1 Rheumatology Unit, AOU Meyer IRCCS, 50139 Firenze, Italy; 2 Rheumatology Unit, Department of Clinical and Experimental Medicine, University of Pisa, 56126 Pisa, Italy; 3 Dentistry and Oral Surgery, AOU Pisana, 56126 Pisa, Italy; 4 Dermatology Unit, AOU Pisana, 56126 Pisa, Italy; 5 Dermatology Unit, AOU Meyer IRCCS, 50139 Firenze, Italy; 6 Neurosciences, Psychology, Drug Research and Child Health (NEUROFARBA) Department, University of Firenze, 50139 Firenze, Italy

**Keywords:** Sjögren’s syndrome, pediatric Sjögren’s disease, ultrahigh-frequency ultrasound, labial salivary glands, childhood-onset Sjögren’s syndrome

## Abstract

Sjögren’s disease (SD) is a chronic autoimmune disease primarily affecting lacrimal and salivary glands. The diagnosis of pediatric SD mostly relies on clinical suspect, resulting in a significant diagnostic delay. Recently, ultrahigh-frequency ultrasound (UHFUS) of labial glands has been proposed as a diagnostic method in adults with suspected SD. Until now, there have been no studies about UHFUS in pediatric diagnostic work-up. The aim of the study was to evaluate the potential role of UHFUS of minor salivary glands in pediatric SD. Consecutive pediatric patients with a diagnosis of pediatric SD seen at AOU Meyer IRCSS were evaluated. Intraoral UHFUS scan of the lip mucosa was performed with Vevo MD equipment, using a 70 MHz probe with a standardized protocol and the images were independently reviewed by two operators. Lip salivary glands were assessed by using a four-grade semiquantitative scoring system for parenchymal alteration and vascularization. Twelve patients were included. When applying UHFUS to this cohort of patients, all patients showed a UHFUS grade of ≥1 with 8/12 showing a mild glandular alteration (i.e., grade 1), 2/12 a moderate glandular alteration (i.e., grade 2) and finally 2/12 a severe glandular alteration (i.e., grade 3). Moderate intraglandular vascularization was seen in 9/12, with only 3/12 showing mild intraglandular vascularization. Due to limited size of the sample, the relationship between histological findings, autoantibodies status and UHFUS grade could not be performed. This preliminary study seems to report UHFUS as feasibility technique to identify salivary gland alterations in children with a clinical suspect of SD.

## 1. Introduction 

Sjögren’s disease (SD) is a chronic autoimmune disease primarily affecting lacrimal and salivary glands. It presents a spectrum of manifestations, ranging from organ-specific autoimmune symptoms to a systemic disorder, and even to an increased risk of B cell lymphoma. Most common symptoms are dry eyes (keratoconjunctivitis sicca) and dry mouth (xerostomia) [1,2].

On the contrary, in children, symptoms of dryness are infrequent due to the low damage burden and the physiological increase in secretory function: recurrent swelling of the parotid glands is commonly reported, but also unspecific symptoms such as arthralgia [1,3,4].

The first official description of a case of pediatric SD was published in 1965 [5], but a description published in 1938 of sicca syndrome and recurrent salivary gland swelling in a 17-year-old girl could represent the first published case of SD in childhood [6].

Nowadays, the diagnosis is established adopting the American College of Rheumatology/European League Against Rheumatism (ACR/EULAR) classification criteria, which are specifically designed for adults [7].

Recently, ultrahigh-frequency ultrasound (UHFUS) of labial glands has been proposed as a novel tool for the noninvasive assessment of labial salivary gland involvement in adults with suspected SD.

Ultrahigh-frequency ultrasound (UHFUS) is a technique, recently introduced, characterized by using ultrasound frequencies in the range between 30 and 100 MHz. Instead, conventional ultrasound techniques involve the use of devices reaching frequencies of 10 to 15 MHz, maximum up to 22 MHz [8,9].

The use of frequencies that are higher than conventional ultrasonography improves spatial resolution at the expense of tissue penetration, which is as low as 10.0 mm from the surface when applying 70 MHz frequencies. However, UHFUS can provide submillimeter image resolution, so it can improve detailed visualizations of superficial anatomical structures [8].

This characteristic has allowed for spreading of the UHFUS technique for the imaging of skin, blood vessels, musculoskeletal anatomy, oral mucosa, and small parts.

In adults, good correlation between ultrasound patterns and histopathologic features of minor salivary glands have been documented, as well as a high negative predictive values of a negative UHFUS in the diagnosis of SD in sicca syndrome in adults [10]. Up to now, no studies have applied UHFUS for the clinical characterization of SD in pediatric patients.

## 2. Objectives

The aim of the study is to identify the ultrasound patterns at UHFUS of minor salivary glands in a cohort of pediatric patients with SD.

## 3. Methods

Consecutive pediatric patients with clinical diagnosis of pediatric-onset SD seen at AOU Meyer between April 2021 and April 2022 were included in this study. All these patients underwent a diagnostic workup comprehensive of minor salivary glands UHFUS.

To be eligible, patients should have received a clinical diagnosis of SD before the age of 16 years, according to a combined set of clinical, serological and instrumental findings. Clinical, radiological and histopathological findings were retrospectively collected using a dedicated case report form (CRF). For each patient, we collected demographics data, age of onset, clinical presentation both at the time of the diagnosis and at the last visit, subjective assessment of ocular, oral and vaginal dryness, serological data including blood count, kidney and liver function, C reactive protein, antinuclear antibodies, anti-Ro/SSA, anti-La/SSB, rheumatoid factor, C3 and C4 levels and presence of hypergammaglobulinemia. Tear secretion was evaluated using the Schirmer test, break-up time (BUT) test or both. We considered levels of <10 mm wetting of the paper strip in Schirmer test and levels <10 sec for BUT test as pathological. Indeed, there no validated parameters for these tests in the healthy pediatric population; however, a recent meta-analysis reported a secretion >15 mm as a normal value, and we assume 10 mm as a cutoff for abnormal secretion [11].

### Ultrasonography and Biopsy

Intraoral UHFUS scan of the lip mucosa was performed with Vevo MD equipment (Vevo^®^ MD, Fujifilm, Visualsonics) with a standardized protocol. For each patient, a standardized intraoral UHFUS examination of the internal surface of the lower lip (central, left and right compartment) was carried out using a 70 MHz probe with the following characteristics: bandwidth 29–71 MHz, nominal frequency 52 MHz, axial resolution 30 μm, lateral resolution 65 μm, maximum depth 10.0 mm, maximum image width 9.7 mm, maximum image depth 10.0 mm, focal depth 5 mm. For each compartment, axial and longitudinal B-mode acquisitions were obtained. The UHFUS scans were performed using a standardized preset, keeping gain, time gain compensation, dynamic range, mechanical index and thermal index constant. Scan depth and focus position were adjusted to optimize the scan. The scans were saved as DICOM format images and were processed using Horos software (https://horosproject.org). The images were independently reviewed by two operators.

Labial salivary glands (LSG) were assessed by using a four-grade semiquantitative scoring system, similar to the OMERACT scoring system used for major salivary glands [8]. Namely, grade = 0: normal glandular parenchyma; grade = 1: the presence of mild glandular alteration, with fine echogenicity in absence of clear alterations, or slight, diffuse glandular hypoechogenicity; grade = 2: a moderate glandular alteration, with the presence of focal hypoechoic areas, but partial conservation of normal glandular parenchyma; and finally, grade = 3: a severe glandular alteration, with diffuse presence of hypoechoic areas in absence of normal glandular parenchyma, or presence of glandular fibrosis. As a second parameter, we evaluated glandular vascularization, using a consensus-based color Doppler semiquantitative score as suggested by Hočevar et al. [12]. It distinguishes four grades: grade 0: no visible vascular signals; grade 1: focal, dispersed vascular signals; grade 2: diffuse vascular signals detected in <50% of the gland; grade 3: diffuse vascular signals in >50% of the gland.

Finally, histopathological parameters were assessed through LSG biopsy, according to Chisholm and Mason scoring system, which includes 5 grades from 0 to 4, based on the presence of slight or moderate lymphocytic infiltration and/or focus of lymphocytes [13,14]). We considered as a positive biopsy any grade of focal sialadenitis, that is, a focus score > 0 foci/4 mm^2^ [3,4,15].

Descriptive statistics were used to summarize the data, including means, medians and standard deviations for continuous variables, and frequencies and percentages for categorical variables.

## 4. Results

We included a total of twelve patients in the study, followed by our center for pediatric SD (*n* = 12, 11 females; 10 Caucasian, 2 Asian). They had a median age at the diagnosis of 13.5 years (range 7.75–17) and a median disease duration of 13.5 months (range 1–94).

Concerning comorbidities, 50% of our patients had also other autoimmune diseases (6/12: 1 celiac disease and Hashimoto thyroiditis, 1 celiac disease, 1 Basedow disease upon Hashimoto thyroiditis, 1 universalis alopecias, 1 systemic lupus erythematosus, 1 autoimmune panniculitis). Five patients also had familiar history positive for autoimmune diseases.

The clinical phenotype at the time of the diagnosis was widely heterogeneous. The features of the patients at time of the diagnosis are summarized in Table 1. Four patients complained of sicca syndrome, primarily affecting the eyes, but in three cases also the mouth; one of them presented not only oral and ocular but also vaginal dryness. They all presented sicca syndrome combined with other symptoms, such as Raynaud phenomenon (2/4), arthritis with morning stiffness (2/4), gastrointestinal manifestations as abdominal pain and/or diarrhea (3/4), asthenia (1/4) and cutaneous vasculitis.

Only two of our patients presented the most typical symptom of pediatric SD, recurrent swelling of parotid gland: one of them is the only boy, the other one is the patient who showed the earliest onset of symptoms (6 years old at the first episode of parotitis). Four patients suffered for cutaneous involvement: one of them presented only a cutaneous lipodystrophy; three patients started with photosensitive erythema, two of them as the only symptom, while in the other one it was combined with kidney involvement (renal tubular acidosis and proteinuria). Two patients started with a generic presentation of arthralgias: one of them combined with systemic symptoms (fever and asthenia), the other one with a mucocutaneous involvement (cutaneous vasculitis and recurring aphthous stomatitis). There was also a girl who only presented Raynaud’s phenomenon.

About laboratory exams, 4/12 patients showed blood count alterations (a variable combination of anemia, lymphocytopenia, neutropenia, thrombocytopenia); 4/12 presented a typical hypergammaglobulinemia (defined as >2 SD according to the age) and 1/12 had hypocomplementemia as the only laboratory alteration. Eleven patients were ANA positive; Ro/SSA were positive in 6/12 while just one tested positive for La/SSB. Two patients presented positive Rheumatoid factor, while all of the patients tested for other autoantibodies (anti-dsDNA or anti-Smith antibodies) resulted negative. However, not all of our patients were tested for each of these antibodies (we had 7/12 tested for Rheumatoid factor, 10/12 for anti-Smith and 8/12 for anti-dsDNA).

In our sample population, 10/12 underwent Schirmer test, with positive results in at least one eye in 7/10, and 8/12 underwent break-up time (BUT) test, with positive results in five cases.

Minor salivary gland biopsy was performed in 9/12, showing inflammatory chronic sialadenitis in 8/12. We chose to avoid performing biopsy in 3/12 patients, since they had typical sicca symptoms combined with positive Ro/SSA autoantibodies.

At the moment of the biopsy, treatment with hydroxychloroquine was ongoing in 11/12; only one of our patients did not assume any therapy.

Only two of our patients (2/12, 16%) met ACR/EULAR diagnostic criteria. Among the 10 patients who did not meet ACR/EULAR criteria, 8/10 (80%) did not present any sicca symptoms (first inclusion criteria), while in the other 2/10 we did not perform all examinations included in classification criteria. We cannot exclude that if we performed more diagnostic exams our patients would have fulfilled diagnostic criteria in a higher percentage. We found more adherence to pediatric Bartunkova criteria (5/12; 41%). In this case, 4/7 (57%) of patients did not fulfil criteria due to a negative autoimmune profile, 2/7 (29%) because of compresence of other autoimmune disease and the other one (1/7, 14%) because she did not present any of the symptoms considered in these criteria (sicca syndrome or systemic symptoms, such as fever of unknown origin, noninflammatory arthralgias, hypokalemic paralysis, abdominal pain).

**Figure 1:**
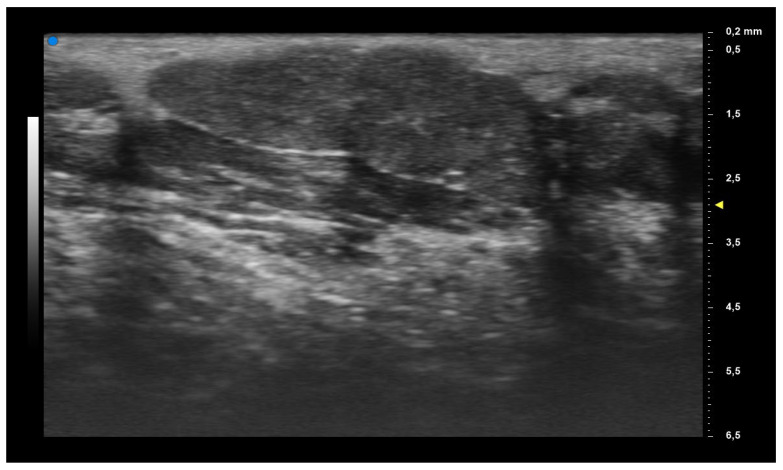
Mild glandular alteration (grade 1).

**Figure 2:**
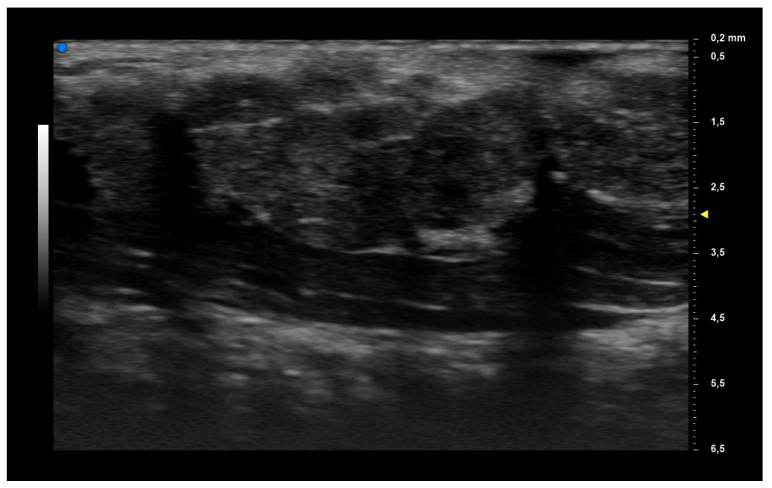
Moderate glandular alteration (grade 2).

**Figure 3:**
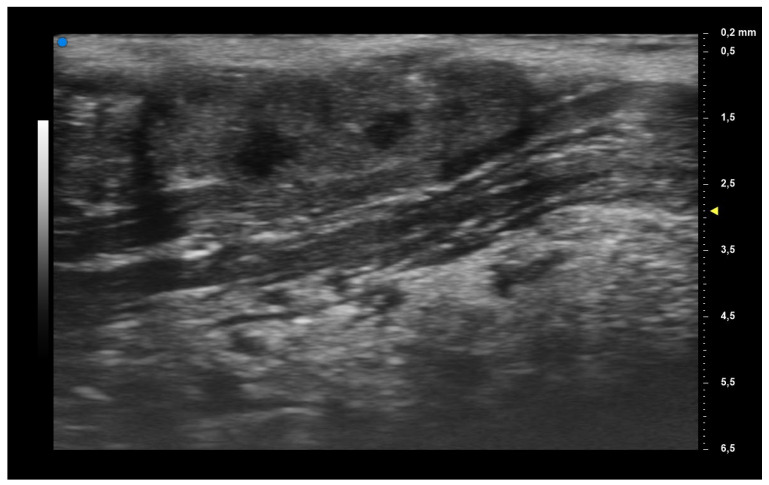
Severe glandular alteration (grade 3).

**Figure 4:**
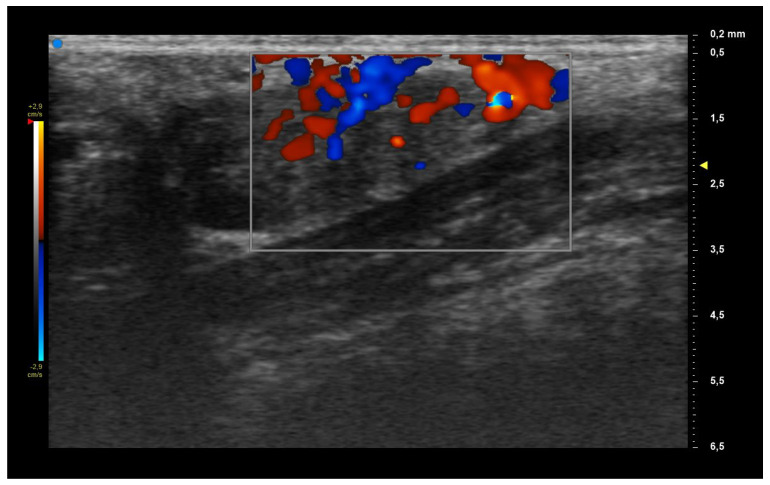
Moderate vascularization.

When applying UHFUS to this cohort of patients, all patients showed a UHFUS grade of ≥1, with 8/12 showing a mild glandular alteration (i.e., grade 1), 2/12 a moderate glandular alteration (i.e., grade 2) and finally 2/12 a severe glandular alteration (i.e., grade 3). Moderate intraglandular vascularization was seen in 9/12, with only 3/12 showing mild intraglandular vascularization. These features are summarized in Table 2. Figure 1 represents grade 1 of parenchymal involvement and it belongs to a patient who presented at the time of diagnosis only photosensitive erythema combined to hypocomplementemia; Figure 2 represents grade 2 of glandular alteration: this patient did not present sicca syndrome nor recurrent swelling of parotid, but she had arthralgias, mucocutaneous and hematological involvement. Figure 3 and Figure 4 are examples of severe glandular alteration with moderate intraglandular vascularization: they belong to a patient with sicca syndrome, mucocutaneous and hematological involvement and presence of Ro/SSA, La/SSB and Rheumatoid factor.

Due to limited size of the sample, the relationship between histological findings, autoantibodies status and UHFUS grade could not be performed. However, through the data at our disposal, we can suppose a correlation between a higher degree of glandular architecture alteration seen at UHFUS and more clinical signs of B cell activation. Indeed, among the two patients with severe glandular alteration, both presented strong hematologic involvement (anemia and/or lymphocytopenia and hypergammaglobulinemia); one of them also had positive LA/SSB and rheumatoid factor, while the other one was the only patient in our sample who also presented renal involvement (proteinuria).

## 5. Discussion

SD is a chronic autoimmune disease characterized clinically by the destruction of the epithelium of the exocrine glands, as a consequence of abnormal B cell and T cell responses to the autoantigens Ro/SSA and La/SSB, among others [16]. One of the main characteristics is the presence of a chronic lymphocytic infiltrate in the glandular parenchyma [9]. The presence of positive autoimmune profile years before diagnosis suggests that the autoimmune process is active years before clinical onset [17]. Due to the multisystem involvement during the course of the disease, clinical heterogeneity in terms of presentation, disease course and outcome is reported for adult patients.

Consequently, there is still no single clinical, laboratory, pathological or radiological feature that could be used as a “gold standard” for its diagnosis and/or classification [2].

The diagnosis of the disease usually is based on objective tests able to quantify patients’ ocular or oral dryness, in association with serologic or histopathologic evidence of an underlying autoimmune basis for the exocrine glandular dysfunction and with demonstration of subsequent inflammation [10].

Diagnosis, therefore, usually combines laboratory exams (i.e., autoantibodies or other markers of B cell activation, such as cytopenia, hypergammaglobulinemia and complement factor consumption), functional tests (i.e., unstimulated saliva flow rate, Ocular Staining Score, Schirmer’s test) and anatomic evaluations, such as salivary glands ultrasound [2,7].

The test still considered the “gold standard” for SD diagnosis remains the LSG biopsy, which allows for identifying a typical focal lymphocytic sialadenitis (FLS), which is considered the hallmark of SD at tissue level [10,18]. LSG have historically been chosen because they are easily accessible, since they lie above the muscle layer, covered by a thin layer of fibrous connective tissue and oral mucous membrane, and because of the low risk of excessive bleeding [13]. However, the biopsy remains an invasive procedure, especially in a pediatric patient and false negative results due to sampling errors.

There have been several proposals for classification criteria that combine the results of these different tests: for a long period, there have been different proposals from different societies, such as 2002 AECG criteria (American–European Consensus Group) [19] or 2012 SICCA-ACR criteria (Sjogren’s International Collaborative Clinical Alliance–American College of Rheumatology) [20], but the SD community recognized the need for an international consensus. Today, the most used is the consensus ACR/EULAR, which results from the overlap of the two previous sets, and it has been recognized by both of the societies [2,7]. However, all of these criteria specifically refer to adult population and they were developed for classification purposes, even though the ACR/EULAR 2016 criteria are also used in clinical practice to establish a diagnosis of SD.

Recently, UFHUS has been proposed as a new method for diagnosis in adults with suspected SD. From the beginning of 2000s, UHFUS was used as a support in dermatologic, vascular, rheumatologic and musculoskeletal fields, mainly in adult populations. There are fewer studies on pediatric populations, mostly concerning vascular morphology [8]. Its importance relies on the capacity to provide a great increase in resolution, that in some case surpasses that of computerized tomography (CT) or magnetic resonance imaging (MRI). The possibility to investigate only the first centimeters of the body surface, which represents the main limit of this method, makes UHFUS particularly suitable for the study of minor salivary glands. Combining its advantages, such as portability, low cost, lack of the need for radiation and sedation and safety, UHFUS can serve as a useful clinical tool in the management of pediatric patients [21].

In patients with suspected SD, minor salivary glands can be imaged with UHFUS by using 70 MHz frequencies, so as to obtain information on parenchymal alterations and on inflammation grade [8]. It can also be used to guide LSG biopsy [9]. According to Ferro et. al, this technique seems to have a higher sensibility than traditional major salivary glands ultrasonography (SGUS) and it is associated with a high negative predictive value. In future, it could be used to avoid biopsy in subject with a low pretest likelihood of being affected by SD, but there is still no defined optimal cutoff [10].

Pediatric SD is a rare condition, and its prevalence is underestimated due to the lack of standardized diagnostic criteria and the subtle early clinical presentation.

Neither the AECG criteria nor the ACR/EULAR criteria are validated for children [3,22]. As already reported, SD has a heterogeneous phenotype, and pediatric subjects have a different clinical presentation. Indeed, they might present reduced prevalence of symptoms related to high disease burden (i.e., symptoms of dryness) as these features are associated with long-lasting inflammation and are more likely to reflect damage accrual more than inflammation itself. Therefore, as the classification criteria are mostly based on features of overt glandular dysfunction, application of these criteria to pediatric patients for a diagnostic purpose might result in underestimation of disease prevalence. Furthermore, tests to assess oral or ocular dryness might be challenging in younger children due to low compliance and due to the lack of normal values for a healthy pediatric population [23]. A proposal for diagnostic criteria in children and adolescents came from Bartunkova et al. [24], but there are still no diagnostic criteria widely accepted for the pediatric population [23].

As suggested by Basiaga et al., lymphocytic infiltration and production of autoantibodies can be interpreted as early features in the pathogenesis that lead to subsequent gland dysfunction and end-organ damage. In line with this hypothesis, children could represent an early stage in the development of SD and the damage accrual over time might result in the adding of clinical features and lead to a full-blown phenotype in later stages of life. This is exemplified by the progressive lymphocytic infiltration of the glands from the pediatric age to adulthood, and so of any focal sialadenitis less than the currently defined cutoff may be sufficient to support diagnosis, as we assumed in our study [3].

Furthermore, a recent study revealed that in pediatric patients with recurrent parotitis, SD diagnosis was very frequent. However, it remains still unclear how to differentiate recurrent juvenile recurrent parotitis from SD, since classification criteria were usually not met [25]. Interestingly, in this setting, SGUS and LSG biopsy were proposed in the workup of recurrent parotitis [25,26]. However, all the patients with parotitis had a positive SGUS and not all the patients had FS higher than 1. Instead, concerning UHFUS, that combine the ultrasonography with the need of labial salivary glands assessment, there are still no studies, but it could represent a complementary tool to differentiate SD patients with or without recurrent parotitis.

All these features make pediatric SD difficult to define, and they cause a significant delay in diagnosis. Improving pediatric diagnosis could not only allow for identifying children with SD prior to gland dysfunction, but also be useful for identifying of classifying adult patients in earlier stages of disease [3].

New approaches to a faster diagnosis are urgently needed in clinical practice. This preliminary pilot study seems to report UHFUS as a feasible technique able to identify salivary gland alterations in children with a clinical suspect of SD. This technique might contribute to driving guided lip biopsy, thus reducing the rate of false negatives.

Limits of our study include the small population sample size, retrospective data collection with some missing data, lack of comparison with healthy patients and the absence of validated parameters in a healthy population for correct understanding of UHFUS results, regarding vascularization in particular. Moreover, we did not perform the UFHUS at a standard time point (i.e., at the diagnosis) and this might thus hamper the possibility of phenotypically stratify these patients; at the same time, the reduced sample size did not allow for making any statistical analysis to correlate UFHUS findings and clinical or histopathological features.

Further studies are currently in progress in our clinics to identify the exact role of UHFUS and its potential predictive role of the various patterns observed in pediatric SD. We would like to primarily define normal patterns of UHFUS in healthy children so as to also establish validated parameters in the pediatric population. Our future studies should include a larger patient sample and comparison with healthy children.

Thanks to this preliminary study, it will be possible to extend the use of a sensible and noninvasive diagnostic method to the pediatric population, thus improving the diagnostic work-up of pediatric SD.

## Figures and Tables

**Table 1:** Clinical and serological features.

Characteristics	Sicca Syndrome (*n* = 4)	Parotitis (*n* = 2)	Non-Sicca, Non-Parotitis (*n* = 6)	Total (*n* = 12)
Age at diagnosis (median year)	14.25	11.5	13.76	13.5
Age at diagnosis (age range)	9.75–17	9.83–13.08	7.75–16	7.75–17
Female, *n* (%)	4 (100%)	1 (50%)	6 (100%)	11 (92%)
Other autoimmune diseases	1 (25%)	2 (100%)	3 (50%)	6 (50%)
Familiar history of autoimmune diseases	1 (25%)	1 (50%)	3 (50%)	5 (42%)
Sicca - Oral dryness - Ocular dryness - Vaginal dryness	3 (75%) 4 (100%) 1 (25%)	0 (0%) 0 (0%) 0 (0%)		3 (25%) 4 (33%) 1/11 (9%)
Parotitis	0 (0%)	2 (100%)		2 (17%)
Arthralgias	0 (0%)	0 (0%)	2 (33%)	2 (17%)
Arthritis	2 (50%)	0 (0%)	0 (0%)	2 (17%)
Asthenia	1 (25%)	0 (0%)	1 (17%)	2 (17%)
Fever	0 (0%)	0 (0%)	2 (33%)	2 (17%)
Raynaud	2 (50%)	0 (0%)	2 (33%)	4 (33%)
Mucocutaneous involvement	1 (25%)	1 (50%)	4 (67%)	6 (50%)
Renal involvement	0 (0%)	0 (0%)	1 (17%)	1 (8%)
Gastrointestinal involvement	3 (75%)	0 (0%)	0 (0%)	3 (25%)
Cytopenia	1 (25%)	0 (0%)	3 (50%)	4 (33%)
Hypergamma	2 (50%)	0 (0%)	3 (50%)	5 (42%)
Hypocomplementemia	0 (0%)	0 (0%)	1 (17%)	1 (8%)
ANA+	4 (100%)	2 (100%)	5 (83%)	11 (92%)
Ro/SSA +	2 (50%)	0 (0%)	4 (67%)	6 (50%)
La/SSB +	1 (25%)	0 (0%)	0 (0%)	1 (8%)

**Table 2:** UHFUS characteristics according to clinical features.

UHFUS Characteristics	Sicca Syndrome (*n* = 4)	Parotitis (*n* = 2)	Non-Sicca, Non- Parotitis (*n* = 6)	Total (*n* = 12)
Grade 1	3 (75%)	1 (50%)	4 (67%)	8 (67%)
Grade 2	0 (0%)	1 (50%)	1 (17%)	2 (17%)
Grade 3	1 (25%)	0 (0%)	1 (17%)	2 (17%)
Mild vascularization	1 (25%)	1 (50%)	1 (17%)	3 (25%)
Moderate vascularization	3 (75%)	1 (50%)	5 (83%)	9 (75%)

## Data Availability

The data presented in this study are available on request from the corresponding author. The data are not publicly available due to privacy reason.
